# Characterization of Heterotrimeric G Protein γ4 Subunit in Rice

**DOI:** 10.3390/ijms19113596

**Published:** 2018-11-14

**Authors:** Sakura Matsuta, Aki Nishiyama, Genki Chaya, Takafumi Itoh, Kotaro Miura, Yukimoto Iwasaki

**Affiliations:** Department of Bioscience and Biotechnology, Fukui Prefectural University, 4-1-1 Kenjojima, Matsuoka, Eiheiji-Town, Fukui 910-1195, Japan; s1873018@g.fpu.ac.jp (S.M.); s1873016@g.fpu.ac.jp (A.N.); s1873012@g.fpu.ac.jp (G.C.); ito-t@fpu.ac.jp (T.I.); miura-k@fpu.ac.jp (K.M.)

**Keywords:** *Dn1-1*, γ-subunit, heterotrimeric G protein, mass spectrometry analysis, RGG4, rice, western blotting

## Abstract

Heterotrimeric G proteins are the molecule switch that transmits information from external signals to intracellular target proteins in mammals and yeast cells. In higher plants, heterotrimeric G proteins regulate plant architecture. Rice harbors one canonical α subunit gene (*RGA1*), four extra-large GTP-binding protein genes (XLGs), one canonical β-subunit gene (*RGB1*), and five γ-subunit genes (tentatively designated *RGG1*, *RGG2*, *RGG3*/*GS3*/*Mi*/*OsGGC1*, *RGG4*/*DEP1*/*DN1*/*qPE9-1*/*OsGGC3*, and *RGG5*/*OsGGC2*) as components of the heterotrimeric G protein complex. Among the five γ-subunit genes, *RGG1* encodes the canonical γ-subunit, *RGG2* encodes a plant-specific type of γ-subunit with additional amino acid residues at the N-terminus, and the remaining three γ-subunit genes encode atypical γ-subunits with cysteine-rich C-termini. We characterized the *RGG4*/*DEP1*/*DN1*/*qPE9-1*/*OsGGC3* gene product Gγ4 in the wild type (WT) and truncated protein Gγ4∆Cys in the *RGG4*/*DEP1*/*DN1*/*qPE9-1*/*OsGGC3* mutant, *Dn1-1*, as littele information regarding the native Gγ4 and Gγ4∆Cys proteins is currently available. Based on liquid chromatography-tandem mass spectrometry analysis, immunoprecipitated Gγ4 candidates were confirmed as actual Gγ4. Similar to α-(Gα) and β-subunits (Gβ), Gγ4 was enriched in the plasma membrane fraction and accumulated in the developing leaf sheath. As *RGG4*/*DEP1*/*DN1*/*qPE9-1*/*OsGGC3* mutants exhibited dwarfism, tissues that accumulated Gγ4 corresponded to the abnormal tissues observed in *RGG4*/*DEP1*/*DN1*/*qPE9-1*/*OsGGC3* mutants.

## 1. Introduction

Heterotrimeric G proteins consist of three subunits (α, β, and γ) in mammals and yeast cells. They act as signal transducers by transferring extracellular information to intracellular components [1,2,3,4]. External signals bind or affect G protein-coupled receptors (GPCRs) to activate them. Activated GPCRs, which function as an intrinsic GDP/GTP exchange factor of GPCRs, convert α-GDP to α-GTP. When GTP binds to the α-subunit (α-GTP), heterotrimeric G proteins dissociate into α subunit (α-GTP) and βγ dimer. The α-subunit and βγ dimer can regulate respective effector molecules. In higher plants, heterotrimeric G proteins are important molecules that regulate plant development and transmit external signals to intracellular target proteins [5,6,7]. Biochemical characteristics of plant heterotrimeric G proteins have been investigated [5]. The signaling mechanisms and effector molecules that regulate plant heterotrimeric G proteins have been reviewed [6], as has the plant morphology of heterotrimeric G protein mutants [7]. *Arabidopsis* harbors three extra-large GTP-binding protein genes (*XLG*s) [8,9], one canonical α-subunit gene (*GPA1*) [10], one canonical β-subunit gene (*AGB1*) [11], and three γ-subunit genes (*AGG1*–*AGG3*) [12,13,14]. Rice (*Oryza sativa*) harbors four extra-large GTP-binding protein genes (*XLG*s) [15], one canonical α-subunit gene (*RGA1*) [16], one canonical β-subunit gene (*RGB1*) [17], and five γ-subunit genes, which are tentatively designated *RGG1*, *RGG2* [18], *RGG3*/*GS3*/*Mi*/*OsGGC1* [19], *RGG4*/*DEP1*/*DN1*/*qPE9-1*/*OsGGC3* [20,21], and *RGG5*/*OsGGC2* [22].

With regard to the γ-subunit genes in *Arabidopsis*, *AGG1* [12] and *AGG2* [13] encode the canonical γ-subunits and *AGG3* [14] encodes the atypical γ-subunit having a cysteine-rich C-terminus. With regard to the γ-subunit genes in rice, *RGG1* encodes the canonical γ-subunit [18], *RGG2* encodes the plant-specific type of γ-subunit [18], and the remaining three γ-subunit genes (*RGG3*/*GS3*/*Mi*/*OsGGC1*, *RGG4*/*DEP1*/*DN1*/*qPE9-1*/*OsGGC3*, and *RGG5*/*OsGGC2*) encode the atypical γ-subunits homologous to *AGG3*. *RGG3* corresponds to *GRAIN SIZE 3* (*GS3*) [19] and *RGG4* corresponds to *DENSE AND ERECT PANICLES 1* (*DEP1*) [20], *DENSE PANICLE1* (*DN1*) [21], and *qPE9-1* [23]. The genome sequence of RGG5 was predicted by Botella [22]. The diversity and agronomical importance of plant γ-subunits have been reviewed previously [24].

Concerning mutants of heterotrimeric G proteins, *xlg1–xlg3* [25], *gpa1* [26], *agb1* [27,28], *agg1* [29], *agg2* [29], and *agg3* [14] in *Arabidopsis*, and *d1* [30,31], Chuan7(GS3-4) and Minghui 63 (GS3-3) [32], *dep1* [20] in rice, have been isolated. By the analysis of *gpa1* [26], *agb1* [28], *d1* [33], and *RGB1* knock-down lines [34], an allele of *GS3* [35], *dep1* [20], it was shown that the plant heterotrimeric G proteins modulated cell proliferation.

Comparing the wild type and mutant responses to external signals, it has been shown that plant heterotrimeric G proteins were involved in transductions of multiple external signals, such as abscisic acid [36,37,38,39,40], auxin [26,28], gibberellin [41,42,43,44], brassinosteroid [26,42,43], sugar [26,45,46], blue light [47,48], ozone [49], elicitors [50,51,52,53]. Plant heterotrimeric G proteins may regulate at integration points for these signals.

Regarding protein–protein interactions in the G protein complex, Klopffleish et al. proposed that 68 highly interconnected proteins form the core G protein interactome in *Arabidopsis*, using the yeast two-hybrid assay (Y2H) [54]. In previous studies, the regulators of G protein signaling protein (AtRGS1) [55], THYLAKOID FORMATION1 (THF1) [46], and cupin domain protein (AtPrin1) [38], were shown to be contained in the interactome. It was also shown that G protein complexes containing Gα subunit—which were solubilized by the plasma membrane—were the huge complexes in rice [18] and *Arabidopsis* [56], respectively. The huge complexes may be a part of the interactome.

Among three atypical γ-subunit genes (*RGG3*, *RGG4*, and *RGG5*) in rice, *RGG3* corresponds to *GRAIN SIZE 3* (*GS3*), which regulates seed length in rice [19,32,35,57,58]. *RGG4* corresponds to *DENSE AND ERECT PANICLES 1* (*DEP1*) [20], *DENSE PANICLE1* (*DN1*) [21], quantitative trait locus *qPE9-1* (*qPE9-1*) [23], which regulate plant architecture including semi-dwarfness, panicle number and panicle erectness. *DEP1* regulates nitrogen-use efficiency in addition to regulating plant architecture [59]. *RGG5* corresponds to *GGC2* [22], which a gene that increases grain length in combination or individually with *DEP1* [57]. These genes are important for rice breeding.

We previously analyzed the native proteins, Gα, Gβ, Gγ1, and Gγ2, localized plasma membrane fraction [18]. However, there is little information on the native proteins translated by *RGG3*, *RGG4*, and *RGG5,* such as Gγ3, Gγ4, and Gγ5, respectively. Among the three atypical γ-subunits, we aimed to identify native Gγ4 and truncated Gγ4 using the anti-Gγ4 domain antibody. The study of the native Gγ4 and truncated Gγ4 is important to understand the function of Gγ4 and truncated Gγ4, which regulate plant architecture. When they are identified, biochemical analysis, namely measurement of subunit stoichiometry and affinity to Gβ, canonical Gα and XLGs, is possible. We tried to identify the native Gγ4 and in wild type rice using an anti-Gγ4 domain antibody. However, the antibody recognized multiple proteins. To identify the native Gγ4 protein, we used the *RGG4* mutant *Dn1-1*, which produces a partially defective protein, as the reference for subtraction. We found candidates of the native Gγ4 and truncated Gγ4 and confirmed that the candidates are actually the native Gγ4 and truncated Gγ4 by liquid chromatography-tandem mass spectrometry (LC-MS/MS) analysis of immunoprecipitation products using anti-Gγ4 domain antibody. The antibody was used to examine the subcellular localization and tissue-specific accumulation of the native Gγ4.

## 2. Results

### 2.1. Rice Heterotrimeric G protein γ4 Gene (RGG4/DEP1/DN1/qPE9-1/OsGGC3) Mutant

To identify the rice heterotrimeric G protein γ4 subunit, Gγ4, we used a mutant possessing the *Dn1-1* mutation in the Nipponbare background. *Dn1-1* displayed characteristics of semi-dwarfism and slightly increased number of spikelets, as described previously [21]. These results indicated that *Dn1-1* mutation clearly affected plant height and panicle number.

### 2.2. Genomic Structure of RGG4 and Protein Structure of Gγ4

**Figure 1:**
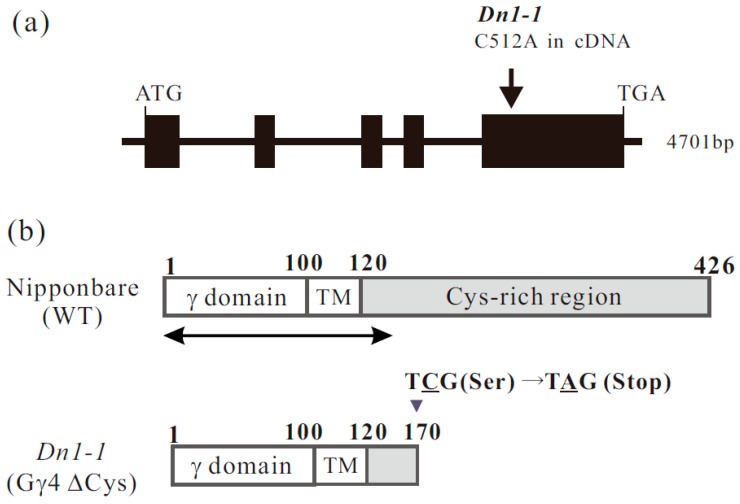
Genome and protein structure of *RGG4*/*DEP1*/*DN1*/*qPE9-1*/*OsGGC3*. (**a**) Genome structure of *RGG4*/*DEP1*/*DN1*/*qPE9-1*/*OsGGC3* and position of the mutation in *RGG4*/*DEP1*/*DN1*/*qPE9-1*/*OsGGC3* mutant *Dn1-1*. The one-base substitution (C512A in full-length cDNA) in *Dn1-1* was in a codon in which TCG (cysteine) was changed to TAG (stop codon). (**b**) Protein structure of the product of *RGG4*/*DEP1/DN1*/*qPE9-1*/*OsGGC3* in wild type (WT) (Gγ4) and *Dn1-1* (Gγ4ΔCys). The canonical γ-domain region is shown as γ domain. Putative transmembrane domain is indicated as TM. The cysteine-rich region is indicated by the gray box. An arrow under WT Gγ4, which covers 137 amino acid residues from the N-terminus, is the region used for recombinant proteins, such as the thioredoxin (Trx)-tagged Gγ4 domain protein (Trx-Gγ4 domain protein), which was used as the antigen, and glutathione S transferase (GST)-tagged Gγ4 domain protein (GST-Gγ4 domain protein), which was used for affinity purification of the antibody.

The genome sequence of *RGG4* was found in RAP-DB (Os09g0441900). We reconfirmed the genome sequence of *RGG4*. *RGG4* consists of five exons (Figure 1a). The translation product, Gγ4, comprises 426 amino acid residues. To prepare recombinant proteins, cDNA for RGG4 was isolated. The molecular weight of Gγ4 calculated from cDNA was 45210 Da. Gγ4 comprised a canonical γ domain of approximately 100 amino acids, a short region with hydrophobic amino acid residues (tentatively termed the transmembrane region, TM), and a region enriched in cysteine residues (Cys-rich region) (Figure 1b).

The *Dn1-1* mutation occurred as a result of a one-base substitution. We reconfirmed the mutation in *Dn1-1* in which C, at position 512 in the full-length cDNA of *RGG4,* was substituted by A (C512A), resulting in the generation of a stop codon (Figure 1a). In *Dn1*-*1*, the mutated protein, tentatively designated Gγ4∆Cys, consisted of 170 amino acid residues (Figure 1b). The cysteine-rich region was absent in Gγ4∆Cys. The molecular weight of Gγ4∆Cys calculated from cDNA was 18997 Da.

### 2.3. Gγ4 Candidates Localized in the Plasma Membrane Fraction

**Figure 2:**
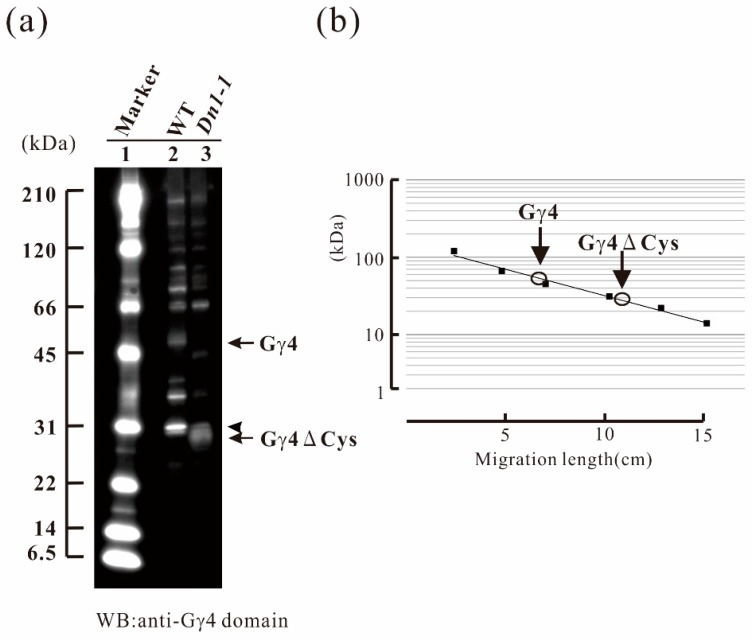
Immunological study of the Gγ4 candidates in leaf sheath of wild type (WT) and *Dn1-1*. (**a**) Plasma membrane protein fractions of WT and *Dn1-1* (10 μg) were used for western blot analysis using anti-Gγ4 domain antibody. Lane 1 contains molecular weight markers. The Gγ4 candidate (indicated by an arrow) was detected as a broad band with a molecular weight of approximately 55 kDa in WT (lane 2). The Gγ4ΔCys candidate (indicated by an arrow) was detected as a broad band with a molecular weight of approximately 27 kDa in *Dn1-1* (lane 3). An arrowhead indicates non-specific bands found in both WT and *Dn1-1*. (**b**) The molecular weights of Gγ4 and Gγ4ΔCys candidates were estimated using molecular weight marker as the standard.

Identification of native Gγ4 and Gγ4∆Cys was carried out using both WT and *Dn1-1* as subtraction references, respectively. As rice Gα and Gβ were known to be localized in the plasma membrane fraction, the plasma membrane fractions of wild type (WT) and *Dn1-1* were prepared using an aqueous two-polymer phase system. Gγ4 candidates were detected by western blotting (WB) using anti-Gγ4 domain antibody. In WT, a 55-kDa protein (Gγ4 candidate) was detected (Figure 2a, lane 2); this band was not observed in *Dn1-1.* The molecular weight of Gγ4 candidate was much higher than that of Gγ4 calculated from WT cDNA (45 kDa). In *Dn1-1*, a 27-kDa protein (Gγ4∆Cys candidate) was detected (Figure 2a, lane 3). The molecular weight of the Gγ4∆Cys candidate was much higher than that of Gγ4∆Cys calculated from cDNA (19 kDa). The molecular weight of Gγ4 and Gγ4∆Cys candidates was measured using molecular weight markers (Figure 2b). Since a sharp band with molecular weight of 31 kDa (indicated by an arrowhead) was found in WT and *Dn1-1*, this band was eliminated to be a Gγ4 candidate.

The chemiluminescent intensity of Gγ4∆Cys was more than 3-fold that of Gγ4, when 10 μg of plasma membrane protein of the WT and *Dn1-1*, respectively, was analyzed by western blot.

### 2.4. Immunoprecipitation of Gγ4, and Gγ4∆Cys Using Anti-Gγ4 Domain Antibody

**Figure 3:**
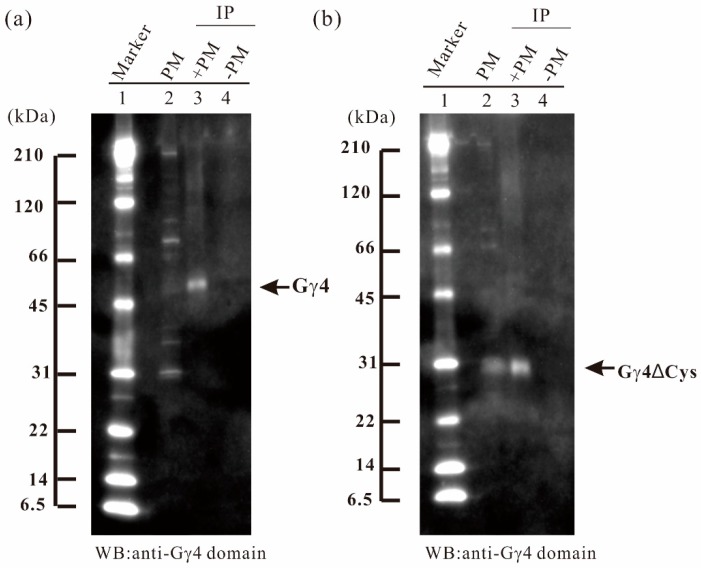
Immunoprecipitation (IP) of Gγ4 and Gγ4ΔCys candidates in leaf sheath of wild type (WT) and *Dn1-1.* (**a**) IP of Gγ4 candidate from solubilized plasma membrane proteins of wild type (WT) using anti-Gγ4 domain antibody. Lane 1, molecular weight markers; lane 2, 10 μg of plasma membrane protein fraction of WT; lane 3, IP product of solubilized plasma membrane proteins and anti-Gγ4 domain antibody; lane 4, control experiment (buffer in the placement of membrane proteins). (**b**) IP of Gγ4ΔCys candidate from solubilized plasma membrane proteins of *Dn1-1* using anti-Gγ4 domain antibody. Lane 1, molecular weight markers; lane 2, 10 μg of plasma membrane protein fraction of *Dn1-1*; lane 3, IP product of solubilized plasma membrane proteins of *Dn1-1* and anti-Gγ4 domain antibody; lane 4, control experiment (buffer in the placement of membrane proteins).

To concentrate Gγ4 and Gγ4∆Cys candidates, immunoprecipitation was carried out using anti-Gγ4 domain antibody. Fifty micrograms of anti-Gγ4 domain antibody was added to 2 mg and 1 mg of solubilized plasma membrane protein of leaf sheath of WT (Figure 3a) and *Dn1-1* (Figure 3b), respectively. Gγ4 and Gγ4∆Cys candidates were collected with the antibody cross-linked Protein A-bound beads. The Gγ4 candidate in WT (55 kDa; Figure 3a, lane 3) and Gγ4∆Cys candidate in *Dn1-1* (27 kDa; Figure 3b, lane 3), were immunoprecipitated. Most other proteins detected by WB of plasma membrane fraction were not observed in the immunoprecipitated products.

### 2.5. LC-MS/MS Analysis

LC-MS/MS analysis was performed to confirm that Gγ4 and Gγ4∆Cys candidates were actually Gγ4 and Gγ4∆Cys and to confirm that proteins with which anti-Gγ4 domain antibody reacted were actually Gγ4 and Gγ4∆Cys. First, we analyzed whether Gγ4 and Gγ4∆Cys candidates in eluate from SDS-PAGE gel pieces of plasma membrane proteins were detected by LC-MS/MS. When the signal intensities of Gγ4 and Gγ4∆Cys in LC-MS/MS were low, we analyzed immunoprecipitation products enriched by anti-Gγ4 domain antibody.

Plasma membrane proteins from WT and *Dn1-1* were analyzed by LC-MS/MS. Forty micrograms each of plasma membrane protein isolated from WT and *Dn1-1* leaf sheath were separated by sodium dodecyl sulfate-polyacrylamide gel electrophoresis (SDS-PAGE) and each lane of the gel was cut into 10 pieces according to molecular weight marker to increase the relative amount of target proteins. After these gel pieces were digested with trypsin, peptides were analyzed by LC-MS/MS in triplicate. WT plasma membrane proteins did not display fragments with *p* < 0.05 Gγ4 and Mascot score of Gγ4 was <50. In LC-MS/MS analysis of *Dn1-1* plasma membrane proteins, five fragments with *p* < 0.05 in Gγ4∆Cys were obtained. The Mascot score in Gγ4∆Cys was 95. Fifteen high accuracy LC-MS/MS fragments of immunoprecipitated WT Gγ4 were obtained (Table 1A). They were chosen as the standard and were numbered. The five fragments from *Dn1-1* plasma membrane protein corresponded to fragment numbers 2, 3, 4, 6, and 7 (Table 1). The Gγ4 candidate was not detected in the LC-MS/MS analysis of WT plasma membrane proteins, and the immunoprecipitation experiment using anti-Gγ4 domain antibody was done.

IP products from WT and *Dn1-1* were separated by SDS-PAGE and analyzed by LC-MS/MS, but the products were not detected by silver staining. In IP products of WT (Figure 3a, lane 3), a gel piece containing a 55-kDa protein was excised and digested with trypsin. The resultant peptides were analyzed by LC-MS/MS. Fifteen Gγ4 fragments with primary mass (*p* < 0.05) were obtained (Table 1A). In IP products of *Dn1-1*, a gel piece containing a 27-kDa protein was excised and digested by trypsin, and the resultant peptides were analyzed by LC-MS/MS. Eight fragments (*p* < 0.05) were obtained (Table 1B).

**Figure 4:**
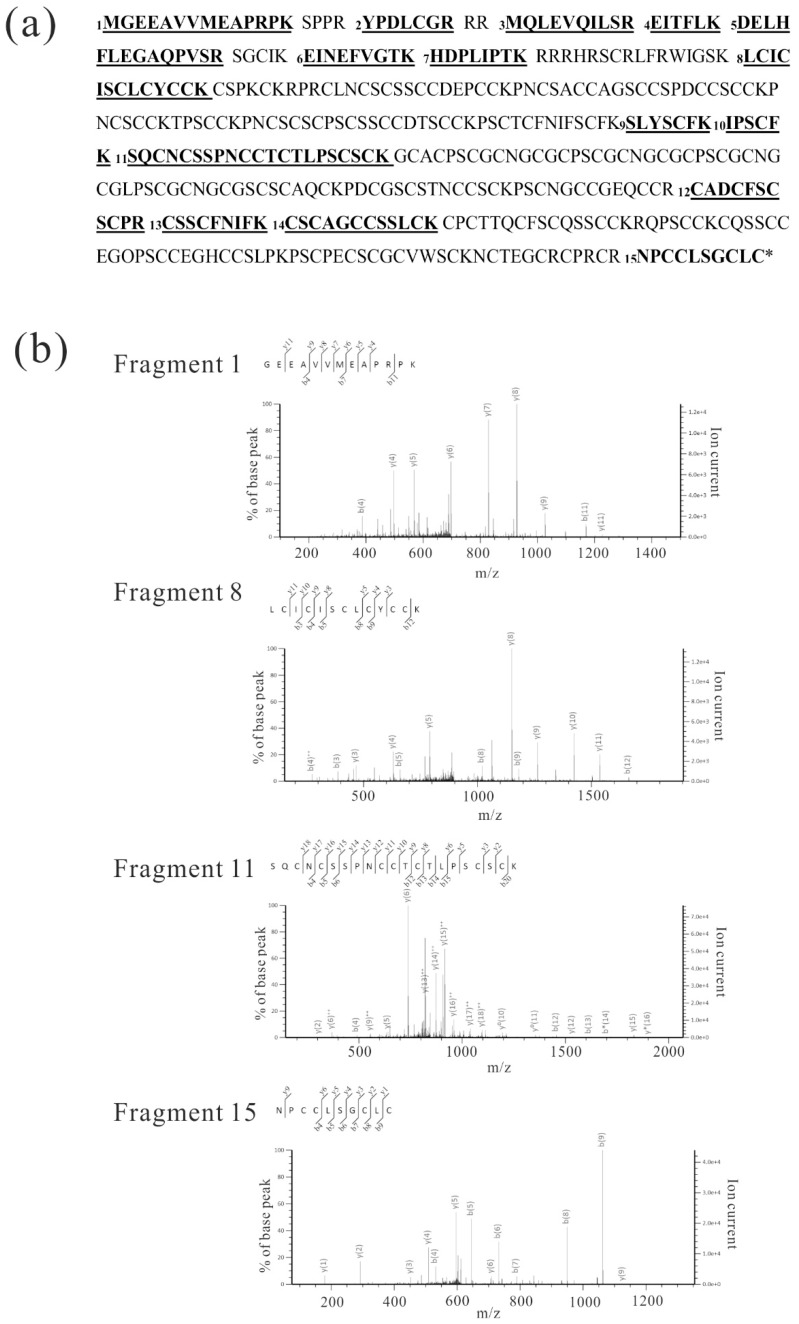
LC-MS/MS analysis of Gγ4 candidates from WT leaf sheath. (**a**) Fifteen peptides (*p* < 0.05) produced by trypsin-digested Gγ4 candidates in wild type (WT) and *Dn1-1* are numbered and underlined in the full-length Gγ4 amino acid sequence. These peptides are listed in Table 1 (A and B). (**b**) MS/MS spectra of four fragments, which were obtained as immunoprecipitation product of Gγ4 in WT (Figure 3a, lane 3). Fragment numbers correspond to Table 1 (A and B).

Figure 4a presents examples of fragments with high accuracy, the MS/MS results of fragments 1, 8, 11, and 15. Based on these results, we concluded that the 55-kDa and 27-kDa proteins were Gγ4 and Gγ4∆Cys, respectively. When the NCBI protein database was used for the analysis of Gγ4 candidates, Gγ4 was found to be annotated using other names, such as ACL27948.1.

### 2.6. Enrichment of Gγ4 and Gγ4∆Cys in the Plasma Membrane Fraction

**Figure 5:**
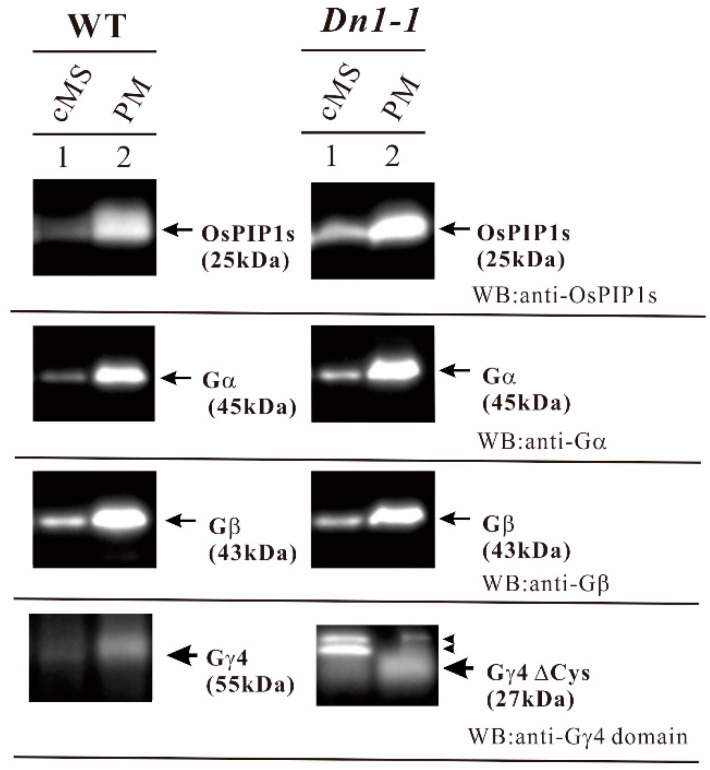
Gγ4 and Gγ4ΔCys in leaf sheath of wild type (WT) and *Dn1-1* were enriched in the plasma membrane fraction (PM). Ten micrograms of each crude microsome fraction (cMS) and plasma membrane (PM) proteins from wild type (WT) and *Dn1-1* were analyzed by western blot using anti-OsPIP1s, anti-Gα, anti-Gβ, and anti-Gγ4 domain antibodies. OsPIP1s is an aquaporin and a plasma membrane marker. OsPIP1s (25kDa), Gα (45kDa), Gβ (43kDa), Gγ4 (55kDa), and Gγ4ΔCys (27kDa) are indicated by arrows. Non-specific bands are indicated by arrowheads.

As LC-MS/MS analysis showed that anti-Gγ4 domain antibody reacted with Gγ4 and Gγ4∆Cys, the amount of Gγ4 and Gγ4∆Cys in the crude microsomal fraction was compared to that in the plasma membrane fraction by western blot (Figure 5). This was in order to check whether Gγ4 and Gγ4∆Cys are enriched in the plasma membrane. To confirm the purity of plasma membrane, the OsPIP1s aquaporin was used as a plasma membrane marker. Gα and Gβ are the subunits of heterotrimeric G protein complex in rice. OsPIP1s, Gα subunit, and Gβ subunit were enriched in the plasma membrane fraction. Gγ4 (55 kDa in WT) and Gγ4∆Cys (27 kDa in *Dn1-1*) were also enriched in the plasma membrane fraction. These results showed that Gγ4 (55 kDa in WT) and Gγ4∆Cys (27 kDa in *Dn1-1*) were localized in the plasma membrane fraction. Non-specific bands (indicated by arrowheads) detected by WB were not analyzed.

### 2.7. Tissue-Specific Accumulation of Gγ4

**Figure 6:**
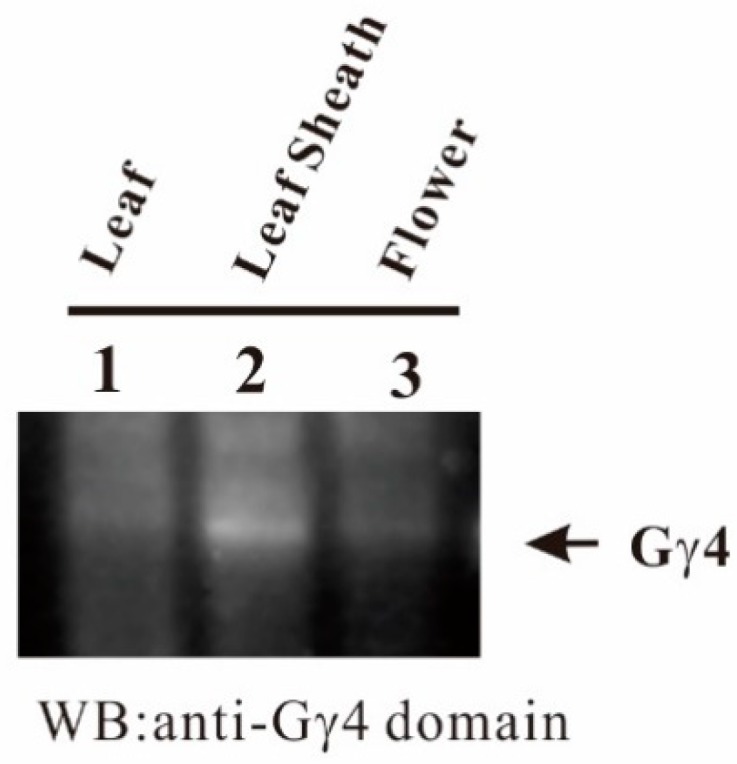
Tissue-specific accumulation of Gγ4 in wild type (WT). Ten micrograms of each plasma membrane protein fraction of leaf, leaf sheath, and flower of WT was analyzed by sodium dodecyl sulfate-polyacrylamide gel electrophoresis and western blotting using anti-Gγ4 domain antibody. Lane 1, leaf from etiolated seedling; lane 2, developing leaf sheath at 8th leaf stage; lane 3, 1–5 cm flower. WB: western blot.

To identify the tissues in which Gγ4 accumulates, the accumulation profile of Gγ4 was studied using plasma membrane fractions of leaf of 1-week-old etiolated seedling, developing leaf sheath, and flowers of WT by western blot. Gγ4 protein predominantly accumulated in the developing leaf sheath (Figure 6).

## 3. Discussion

Rice has three atypical γ-subunit genes (*RGG3*, *RGG4*, and *RGG5*) in the heterotrimeric G protein complex, which are homologous to *AGG3*. Both *RGG3* regulating seed length and *RGG4* regulating plant architecture including semi-dwarfness, panicle number and panicle erectness, respectively, are important genes for breeding. In this study, we aimed to identify the native protein translated by *RGG4*/*DEP1*/*DN1*/*qPE9-1*/*OsGGC3.* Identification of the native Gγ4 and truncated Gγ4 is important, to understand the function of *RGG4*.

First, we detected the Gγ4 candidate from WT and truncated Gγ4 candidate Gγ4∆Cys from *Dn1-1* by WB using anti-Gγ4 domain antibodies (Figure 2). SDS-PAGE estimated the molecular weights of Gγ4 and Gγ4∆Cys candidates as 55 and 27 kDa, respectively, which were higher than the molecular mass calculated using cDNAs (45 and 19 kDa, respectively). These results indicated that modifications, such as glycosylation, ubiquitination, phosphorylation, lipid modification, which include palmitoylation etc., might have occurred after translation in Gγ4 and Gγ4∆Cys candidates. As Gγ4 and Gγ4∆Cys were detected as broad bands by WB, some modification may have occurred. Identification of the modifications will be the subject of future studies. When 10 μg of plasma membrane protein from each of the WT and *Dn1-1* developing leaf sheath were analyzed by western blot, the chemiluminescent intensity of Gγ4∆Cys was more than 3-fold that of Gγ4 (Figure 2). The reason that the amount of Gγ4 was less than that of Gγ4∆Cys may be that Gγ4 is degraded by proteases. Another possibility is that Gγ4∆Cys accumulates in the plasma membrane with other proteins, including Gβ.

To obtain concrete evidence of whether the Gγ4 and Gγ4∆Cys candidates detected by WB were actually Gγ4 and Gγ4∆Cys proteins, IP products of Gγ4 and Gγ4∆Cys candidates were analyzed by LC-MS/MS (Figure 3 and Figure 4). Fifteen fragments displayed *p* < 0.05 using the Mascot search engine were obtained from the two candidates. These results indicated that the Gγ4 and Gγ4∆Cys candidates were indeed Gγ4 and Gγ4∆Cys, respectively.

*Dn1-1* displays semi-dwarfness and increased spikelet number [21]. Thus, Gγ4 regulates plant architecture. Gγ4 and Gγ4∆Cys accumulated in the plasma membrane fraction of developing leaf sheath (Figure 5). The tissue in which Gγ4 and Gγ4∆Cys accumulated corresponded to the tissue that exhibited semi-dwarfness in *Dn1-1* (Figure 6). Although *Dn1-1* exhibited slightly increased number of spikelets [21], the amount of Gγ4 in flower of 1–5 cm in length was less than that in the developing leaf sheath (Figure 6). In the RAP-database, an accumulation profile of Gγ4 mRNA by microarray analysis was found. The relative amount of Gγ4 mRNA in the flower was similar to that in the leaf sheath. The accumulation profile of Gγ4 protein seems to be slightly different from that of Gγ4 mRNA. The accumulation of Gγ4 protein in flowers may be limited to some specific stage and/or organ, such as the inflorescent meristem, in which other proteins necessary for stable accumulation of Gγ4 protein may be present.

Sun et al. studied the interaction between DEP1 (Gγ4 in this study) and Gβ using yeast two-hybrid (Y2H) and bimolecular fluorescence complementation (BiFC) methods [59]. The G protein γ-like domain (GGL) of DEP1 was necessary for binding to Gβ. It is considered that approximately 90–100 N-terminal amino acid residues comprise the canonical γ-domain and the subsequent 20 amino acid residues comprise a transmembrane domain in DEP1. As the truncated proteins of *dep1-1* [59] and *Dn1-1* in this study [21] comprised 196 and 170 amino acid residues, respectively, they can bind to Gβ and anchor in the plasma membrane. Sun et al. also studied the subcellular localization of DEP1 and Gβ by BiFC. DEP1 interaction with Gβ on the plasma membrane was revealed. The truncated protein of *dep1-1* also interacted with Gβ on the plasma membrane. In this research, Gγ4 and Gγ4∆Cys were enriched in rice plasma membrane, as was Gβ. Our results corroborate the findings of Sun et al. As native Gγ4 and Gγ4∆Cys have canonical γ and transmembrane domains, these proteins may form a dimer with Gβ and might anchor on the plasma membrane.

Sun et al. indicated that DEP1 is localized in the nucleus, in addition to the plasma membrane [59]. Taguchi-Shiobara et al. also demonstrated that DN1 (DEP1) is localized in the nucleus by analysis of green fluorescence protein–Gγ4 fusion protein [21]. Although we detected faint broad bands in the 2000× *g* precipitate, which may correspond to Gγ4, it was not clear whether these broad bands were actually Gγ4 or not. If Gγ4 was localized in the nucleus, its amount was less than that in the plasma membrane fraction. Although we purified our antibodies using affinity purification method, our antibody recognized Gγ4 and other proteins in WB (Figure 2 and Figure 5). Antibody production using another part of Gγ4 may be necessary to prepare a high-specificity antibody.

Concerning G protein signaling, the unusual βγ dimer composed of GβGγ4∆Cys may be the cause of the shortened plant height and increased spikelet number. In fact, the relative amount of Gγ4∆Cys is much higher than that of Gγ4 in the developing leaf sheath. It will be interesting to determine the function of the unusual βγ dimer (GβGγ4∆Cys) with reference to the G protein signaling model [5,6].

Gβ subunits interact with Gγ subunits and subsequently the βγ dimer was formed in mammals and yeast [1,2,3,4]. It has also been shown in plants that Gβ subunits interact with Gγ subunits, using pull down assay [12,13], Y2H [13,18], the split-ubiquitin system [14], bimolecular fluorescence complementation assay (BiFC) [57,59], etc. We confirmed that Gγ4 and Gγ4∆Cys interacted with Gβ using Y2H (data not shown).

Cell number of stem in longitudinal axis is higher in NIL-dep1 [20]. Gγ4 also modulate cell proliferation, similar to Gα [33], Gβ [34], and Gγ3 [35]. It will be important to research the mechanism of cell proliferation which the G protein subunits regulate.

Candidates of both βγ1 and βγ2 dimers present two different fractions in gel filtration, with the former evident as huge complexes containing both βγ1 and βγ2 dimers, and the latter being the dissociated form of the huge complex as a sole βγ1 or βγ2 dimer, in the plasma membrane of etiolated rice seedling [18]. Although this may have resulted from artificial dissociation during solubilization and gel fractionation, this approach will be important for understanding the heterotrimeric G protein complex. As we identified native Gγ4 and Gγ4∆Cys in this study, it will be possible to determine whether Gγ4 is a component of the heterotrimeric G protein complex containing canonical Gα and XLGs.

Kunihiro et al. showed that rice DEP1 (Gγ4) may function as a trap for cadmium ions on yeast cells and *Arabidopsis* [60]. This study gives a new insight into enzymatic function of rice DEP1 (Gγ4). The comparison of cadmium ions between wild type and *Dn1-1* may be helpful for further study of G protein signaling.

## 4. Materials and Methods

### 4.1. Plant Materials

A rice cultivar (*O. sativa* L. cv. Nipponbare) and a heterotrimeric G protein γ4 mutant (*Dn1-1*) [21] were used in this study. All rice plants were grown under 14-h light (50,000 lux and 28 °C) and 10-h dark (25 °C) cycle or under natural field condition. Nipponbare is abbreviated as WT in the manuscript.

### 4.2. Sequencing and Confirmation of RGG4

Genomic DNA was isolated from whole plants of WT and *Dn1-1* by an extraction method using cetyltrimethylammonium bromide [61]. Using this DNA as the template, PCR was performed using >20 sets of PCR primers to cover 4701 bases of *RGG4* (Os09g0441900). The amplified DNA fragments were sequenced directly using the same primers that were used for amplification.

### 4.3. RNA Isolation, Reverse Transcription, and cDNA Encoding of the Heterotrimeric G Protein γ4 Subunit

Total RNA from flower tissue was directly extracted using RNeasy Plant Mini Kits (Qiagen). The first strand of cDNA was synthesized using Super Script First Strand Synthesis System for RT-PCR (Invitrogen, Carlsbad, CA, USA). Total RNA (0.5 μg) and oligo-dT were used as the template and primer, respectively, for first strand cDNA synthesis. To isolate *RGG4* cDNA, primers were designed based on the database information (Os03g0407400): RGG4 forward: 5′ gtggttctgagttggccgtt 3′ and RGG4 reverse: 5′ caaccaaaaaaggatctagatc 3′. The amplified PCR products were sub-cloned into pCR4 (Invitrogen) and sequenced with a THERMO sequence dye terminator cycle sequencing kit (Amersham Biosciences, Little Chalfont, UK) using a model 377 DNA sequencer (Applied Biosystems, Foster City, CA, USA).

### 4.4. Preparation of cMS and Plasma Membrane Fractions of Rice

cMSs fraction of WT were prepared from etiolated seedlings grown for 5 days at 28 °C, from developing leaf sheaths at the 8th leaf stage, and from 1–5 cm flowers as described previously [18]. In *Dn1-1*, the cMS fraction from developing leaf sheaths at the 8th leaf stage was prepared. All procedures for membrane preparation were performed at 4 °C. Tissue homogenate was centrifuged at 10,000× *g* for 10 min, and the resultant supernatant was centrifuged at 100,000× *g* for 1 h. The precipitate (100,000× *g* precipitate) was designated the crude microsomal fraction (cMS). Plasma membrane fractions were prepared from cMS using an aqueous two-polymer phase system [62].

### 4.5. SDS-PAGE

Electrophoresis was performed on 12.5% and 10%/20% gradient polyacrylamide gels containing 0.1% SDS as described previously [63].

### 4.6. Preparation of Trx-Gγ4 and GST-Gγ4 Domain Proteins

cDNA encoding 137 amino acid residues from the N-terminal of rice Gγ4 protein was amplified by PCR using primers. The cDNA contained the Gγ4 domain and putative transmembrane region: RGG4 domain forward: 5′ ccatggctcatatggatatcatgggggaggaggcggtggtg 3′ and RGG4 domain reverse: 5′ aagcttcccgggtcaactgcagtttggcttacagcatg 3′. Amplified cDNA was sub-cloned in pCR4 (Invitrogen) and the fragment containing the Gγ4 domain was digested with *Eco*RV and *Hind*III. The fragment was sub-cloned in pET32a containing thioredoxin (Trx) and histidine (His) tags (Novagen, Madison, WI, USA). The resultant clone, Trx-Gγ4 domain vector, was transformed in T7 Express *lysY/I^q^ Escherichia coli* (New England Biolabs, Ipswich, MA, USA). The recombinant protein was designated Trx-Gγ4 domain protein. cDNA containing the Gγ4 domain was also sub-cloned in pET41 containing glutathione S transferase (GST) and histidine (His) tags (Novagen). The resultant clone, GST-Gγ4 domain vector, was transformed in T7 Express *lysY/I^q^ E. coli* (New England Biolabs). The recombinant protein was designated as GST-Gγ4 domain protein.

The overexpression of Trx-Gγ4 domain protein and GST-Gγ4 domain protein in T7 Express *lysY*/*I^q^ E. coli* was carried out as previously described [63]. Induction was performed at 37 °C. Induction was started by addition of isopropyl β-d-1-thiogalactopyranoside (final concentration, 1 mM). After 3 h, *E. coli* was harvested at 10,000× *g* for 5 min at 4 °C and stored at −80 °C until required.

As the Trx-Gγ4 domain protein and GST-Gγ4 domain protein were inclusion bodies, both proteins were solubilized in 6 M guanidine hydrochloride, 10 mM Tris HCl, pH 8.0. Solubilized proteins were applied to Ni-NTA agarose (Qiagen, Hilden, Germany). Purification of both proteins was performed according to the protocols recommended by the manufacturers.

The antibody was raised against Trx-Gγ4 domain protein in rabbits. Affinity purification of the antibody was carried out using a polyvinylidene fluoride (PVDF) membrane (Millipore, Burlington, MA, USA) immobilized with GST-Gγ4 domain protein.

### 4.7. Western Blotting (WB)

Proteins were separated by 12.5% or 10/20% gradient SDS-PAGE, and blotted on a PVDF membrane (Millipore). Antibody against rice Gγ4 domain was affinity-purified in this study. Antibodies against rice heterotrimeric G protein α- and β-subunits (anti-Gα and anti-Gβ antibodies, respectively) were used as described previously [18]. Antibody against the aquaporin plasma membrane marker (anti-OsPIP1s) was purchased from Operon Biotechnologies. The Chemi-Lumi One Markers Kit (Nacalai Tesque, Kyoto, Japan) was used as the molecular weight marker for WB. Affinity-purified anti-Gγ4 domain antibody was used at 5 μg IgG/mL for WB. Anti-Gα and anti-Gβ antibodies were used at 1 μg IgG/mL for WB. Anti-OsPIP1s was diluted as described by the manufacture.

ECL™ peroxidase labeled anti-rabbit secondary antibody was purchased from GE Healthcare (Little Chalfont, UK). ECL Immobilon Western Chemiluminescent HRP Substrate (Millipore) was used for detection reagent for WB. The chemiluminecent signal was measured by Fusion SL (M&S Instruments, Orpington, UK).

### 4.8. Immunoprecipitation (IP)

Fifty micrograms of affinity-purified anti-Gγ4 domain antibody was bound to 50 mg of Protein A-bound magnetic beads (Millipore, Burlington, MA, USA). After washing twice with 1× PBS, anti-Gγ4 domain antibody and Protein A were cross-linked with dimethyl pimelimidate dihydrochloride (DMP). The conditions followed for cross-linking were according to the protocols recommended by the manufacturer. After quenching, the magnetic cross-linked beads with anti-Gγ4 domain antibody were stored at 4 °C until use.

SDS (0.1 mL of a 10% solution) was added to 0.9 mL of the plasma membrane fraction (1 mg protein/10 mg SDS/mL) and denatured for 5 min at 90 °C. After diluting the solubilized fraction with 10 mL of 1× Tris-buffered saline containing 1% Tween-20, magnetic beads cross-linked with 50 μg of anti-Gγ4 domain antibody were added. After incubation for 2 h at 25 °C, the magnetic beads were collected into a 1.5-mL tube and washed three times each with 0.5 mL of 1× TBS containing 0.1% Tween-20 and 0.5 mL of 1× TBS. Proteins were eluted from the beads using 40 μL of dissociation buffer (Bio-Rad) without the reducing agent. Eight microliters of each eluate was used for LC-MS/MS.

### 4.9. Protein Reduction, Alkylation, and Trypsin Digestion for LC-MS/MS Analysis

For LC-MS/MS analysis, 40 μg of leaf sheath plasma membrane proteins from WT and *Dn1-1* were analyzed using 15% SDS-PAGE. Electrophoresis was stopped at a position where bromophenol blue was 3 cm away from the stacking gel. The 3-cm long gel was excised in 10 pieces according to the molecular weight marker, Precision Plus Protein™ Kaleidoscope™ (Bio-Rad Laboratories, Hercules, CA, USA) without staining. These gel pieces were subjected to trypsin digestion. In some cases, gels were silver stained using Pierce Silver Stain for Mass Spectrometry (Thermo Fisher Scientific, Waltham, MA, USA).

Gel pieces were resuspended in 50 mM NH_4_HCO_3_, reduced with 50 mM dithiothreitol for 30 min at 56 °C, and alkylated with 50 mM iodoacetamide for 30 min at 37 °C in the dark. Alkylated proteins in the gels were digested with 10 μg/mL trypsin solution (Promega, Madison, WI, USA) for 16 h at 37 °C. The resultant peptides were concentrated and suspended in 0.1% formic acid and analyzed by LC-MS/MS.

### 4.10. Protein Identification Using Nano LC-MS/MS

The peptides were loaded onto the LC system (EASY-nLC 1000; Thermo Fisher Scientific) equipped with a trap column (EASY-Column, C18-A1 5 µm, 100 µm ID × 20 mm; Thermo Fisher Scientific), equilibrated with 0.1% formic acid, and eluted with a linear acetonitrile gradient (0–50%) in 0.1% formic acid at a flow rate of 200 nL/min. The eluted peptides were loaded and separated on a column (C18 capillary tip column, 75 µm ID × 120 mm; Nikkyo Technos, Tokyo, Japan) with a spray voltage of 1.5 kV. The peptide ions were detected using MS (LTQ Orbitrap Elite MS; Thermo Fisher Scientific) in data-dependent acquisition mode with the installed Xcalibur software (version 2.2; Thermo Fisher Scientific). Full-scan mass spectra were acquired in MS over 400–1500 *m*/*z* with a resolution of 60,000. The 10 most intense precursor ions were selected for collision-induced fragmentation in the linear ion trap at normalized collision energy of 35%. Dynamic exclusion was employed within 90 s to prevent repetitive selection of peptides.

### 4.11. MS Data Analysis

Protein identification was performed using the Mascot search engine (version 2.5.1, Matrix Science, London, UK) and the in-house database constructed using the amino acid sequences of rice heterotrimeric G protein subunits. For both searches, carbamidomethylation of cysteine was set as the fixed modification and oxidation of methionine was set as a variable modification. Trypsin was specified as the proteolytic enzyme, and one missed cleavage was allowed. Peptide mass tolerance was set at 10 ppm, fragment mass tolerance was set at 0.8 Da, and peptide charges were set at +2, +3, and +4. An automatic decoy database search was performed as a part of the search. Mascot results were filtered with the Percolator function to improve the accuracy and sensitivity of peptide identification. The minimum requirement for identification of a protein was two matched peptides. Significant changes in the abundance of proteins between samples were determined (*p* < 0.05).

### 4.12. Gene ID

The accession number of rice heterotrimeric G protein α, β, and γ4 subunit genes (*RGA1*, *RGB1*, and *RGG4*, respectively) is Os05g0333200, Os03g0669200, and Os09g0441900, respectively.

## Figures and Tables

**Table 1:** LC-MS/MS analysis of Gγ4 fragments in leaf sheath of wild type (WT) and *Dn1-1.* (**A**) Gγ4 fragments in immunoprecipitation products of the WT membrane fraction. (**B**) Gγ4 fragments in immunoprecipitation products of the *Dn1-1* membrane fraction.

(**A**)					
**Fragments**	**Observed**	**Mr (Expt)**	**Mr (Calc)**	**Expect**	**Peptide**
1	706.8601	1411.7057	1411.7129	1.80 × 10^−6^	M.GEEAVVMEAPRPK.S
2	440.7008	879.3871	879.3909	0.0015	R.YPDLCGR.R
3	608.8365	1215.6585	1215.6645	9.20 × 10^−7^	R.MQLEVQILSR.E
4	375.7118	749.409	749.4323	0.0075	R.EITFLK.D
5	799.3979	1596.7812	1596.7896	1.20 × 10^−6^	K.DELHFLEGAQPVSR.S
6	1036.5248	1035.5175	1035.5237	2.10 × 10^−5^	K.EINEFVGTK.H
7	460.7616	919.5086	919.5127	0.0077	K.HDPLIPTK.R
8	905.3625	1808.7105	1808.721	7.50 × 10^−7^	K.LCICISCLCYCCK.C
9	904.4171	903.4098	903.416	0.0006	K.SLYSCFK.I
10	751.3766	750.3693	750.3734	0.00025	K.IPSCFK.S
11	856.6377	2566.8913	2566.909	66.70 × 10^−8^	K.SQCNCSSPNCCTCTLPSCSCK.G
12	710.2448	1418.4751	1418.4836	1.30 × 10^−8^	R.CADCFSCSCPR.C
13	581.7522	1161.4898	1161.4947	3.90 × 10^−7^	R.CSSCFNIFK.C
14	725.2517	1448.4889	1448.4975	7.00 × 10^−7^	K.CSCAGCCSSLCK.C
15	620.7291	1239.4437	1239.4505	1.10 × 10^−5^	R.NPCCLSGCLC
(**B**)					
**Fragments**	**Observed**	**Mr (expt)**	**Mr (Calc)**	**Expect**	**Peptide**
1	706.8604	1411.7063	1411.7129	0.000016	M.GEEAVVMEAPRPK.S
2	440.7009	879.3872	879.3909	0.002	R.YPDLCGR.R
3	608.8362	1215.6579	1215.6645	5.6 × 10^−7^	R.MQLEVQILSR.E
4	375.7219	749.4292	749.4323	0.0019	R.EITFLK.D
5	799.3994	1596.7842	1596.7896	0.000046	K.DELHFLEGAQPVSR.S
6	1036.5227	1035.5154	1035.5237	0.000024	K.EINEFVGTK.H
7	460.7607	919.5068	919.5127	0.018	K.HDPLIPTK.R
8	905.3626	1808.7107	1808.721	0.0000015	K.LCICISCLCYCCK.C

Eight microliters of each eluate in immunoprecipitates of WT and *Dn1-1* (A and B) were used for LC-MS/MS. Fragments of trypsin-digested Gγ4 candidates (*p* < 0.05) are shown. Fragment numbers correspond to Figure 4a. Mr (expt) and Mr (calc) correspond to the theoretical molecular mass and molecular mass that was calculated from the observed molecular mass, respectively. The scores by Mascot search were 859 (A) and 505 (B) for WT and *Dn1-1*, respectively.

## Abbreviations

*agb1*	Mutant of heterotrimeric G protein β subunit gene in Arabidopsis
*agg1*	Mutant of heterotrimeric G protein γ1 subunit gene in Arabidopsis
*agg2*	Mutant of heterotrimeric G protein γ2 subunit gene in Arabidopsis
*agg3*	Mutant of heterotrimeric G protein γ3 subunit gene in Arabidopsis
*AGB1*	Heterotrimeric G protein β subunit gene in Arabidopsis
*AGG1*	Heterotrimeric G protein γ1 subunit gene in Arabidopsis
*AGG2*	Heterotrimeric G protein γ2 subunit gene in Arabidopsis
*AGG3*	Heterotrimeric G protein γ3 subunit gene in Arabidopsis
*d1*	Mutant of heterotrimeric G protein α subunit gene in rice
*DEP1*	DENCE AND ERRECT PANICLES 1
*DN1*	DENCE PANICLE 1
*gpa1*	Mutant of heterotrimeric G protein α subunit gene in Arabidopsis
Gα	Heterotrimeric G protein α subunit
Gβ	Heterotrimeric G protein β subunit
Gγ	Heterotrimeric G protein γ subunit
*GPA1*	Heterotrimeric G protein α subunit gene in Arabidopsis
*GS3*	GRAIN SIZE 3 gene
IP	Immunoprecipitation
*OsGGC1*	A gene of heterotrimeric G protein γ subunit Type-C in rice, which corresponds to *GS3/RGG3*
*OsGGC2*	A gene of heterotrimeric G protein γ subunit Type-C in rice, which corresponds to *RGG5*
*OsGGC3*	A gene of heterotrimeric G protein γ subunit Type-C in rice, which corresponds to which corresponds to *DEP1/RGG4*
PM	Plasma membrane
*qPE9-1*	A quantitative trait locus regulating plant architecture including panicle erectness in rice
*RGA1*	Heterotrimeric G protein α subunit gene in rice
*RGB1*	Heterotrimeric G protein β subunit gene in rice
*RGG1*	Heterotrimeric G protein γ1 subunit gene in rice
*RGG2*	Heterotrimeric G protein γ2 subunit gene in rice
*RGG3*	Heterotrimeric G protein γ3 subunit gene in rice
*RGG4*	Heterotrimeric G protein γ4 subunit gene in rice
*RGG5*	Heterotrimeric G protein γ5 subunit gene in rice
WB	Western blot
WT	Wild-type
*XLG*	A gene coding extra-large GTP-binding protein
XLG	Extra-large GTP-binding protein
*xlg*	Mutant of a gene coding extra-large GTP-binding protein
